# The Systems Analysis and Improvement Approach: specifying core components of an implementation strategy to optimize care cascades in public health

**DOI:** 10.1186/s43058-023-00390-x

**Published:** 2023-02-14

**Authors:** Sarah Gimbel, Kristjana Ásbjörnsdóttir, Kristin Banek, Madeline Borges, Jonny Crocker, Joana Coutinho, Vasco Cumbe, Aneth Dinis, McKenna Eastment, Douglas Gaitho, Barrot H. Lambdin, Stephen Pope, Onei Uetela, Carmen Hazim, R. Scott McClelland, Ana Olga Mocumbi, Alberto Muanido, Ruth Nduati, Irene N. Njuguna, Bradley H. Wagenaar, Anjuli Wagner, George Wanje, Kenneth Sherr

**Affiliations:** 1 Department of Child, Family, and Population Health Nursing, University of Washington, Magnuson Health Science Bldg, Seattle, WA USA; 2 Department of Global Health, University of Washington, Seattle, WA USA; 3 Center for Public Health Sciences, University of Iceland, Reykjavík, Iceland; 4 Department of Epidemiology, University of Washington, Seattle, WA USA; 5 Institute for Global Health and Infectious Diseases, University of North Carolina, Chapel Hill, NC USA; 6 Comité para a Saúde de Moçambique, Beira, Mozambique; 7 Ministry of Health, Provincial Health Department, Sofala, Mozambique; 8 Ministry of Health, National Department of Public Health, Maputo, Mozambique; 9 Department of Medicine, University of Washington, Seattle, WA USA; 10 Network of AIDS Researchers of East and Southern Africa, Nairobi, Kenya; 11 RTI International, Berkeley, CA USA; 12 Instituto Nacional de Saúde de Maputo, Maputo, Mozambique; 13 Universidade Eduardo Mondlane, Maputo, Mozambique; 14 University of Nairobi, Nairobi, Kenya; 15 Research and Programs, Kenyatta National Hospital, Nairobi, Kenya; 16 Department of Medical Microbiology, University of Nairobi, Nairobi, Kenya

**Keywords:** Systems Analysis and Improvement Approach (SAIA), Systems engineering, Cascade analysis, Process mapping, Continuous quality improvement, Implementation science, Implementation outcomes, ERIC strategies, Implementation strategy, Implementation strategy specification

## Abstract

**Background:**

Healthcare systems in low-resource settings need simple, low-cost interventions to improve services and address gaps in care. Though routine data provide opportunities to guide these efforts, frontline providers are rarely engaged in analyzing them for facility-level decision making. The Systems Analysis and Improvement Approach (SAIA) is an evidence-based, multi-component implementation strategy that engages providers in use of facility-level data to promote systems-level thinking and quality improvement (QI) efforts within multi-step care cascades. SAIA was originally developed to address HIV care in resource-limited settings but has since been adapted to a variety of clinical care systems including cervical cancer screening, mental health treatment, and hypertension management, among others; and across a variety of settings in sub-Saharan Africa and the USA. We aimed to extend the growing body of SAIA research by defining the core elements of SAIA using established specification approaches and thus improve reproducibility, guide future adaptations, and lay the groundwork to define its mechanisms of action.

**Methods:**

Specification of the SAIA strategy was undertaken over 12 months by an expert panel of SAIA-researchers, implementing agents and stakeholders using a three-round, modified nominal group technique approach to match core SAIA components to the Expert Recommendations for Implementing Change (ERIC) list of distinct implementation strategies. Core implementation strategies were then specified according to Proctor’s recommendations for specifying and reporting, followed by synthesis of data on related implementation outcomes linked to the SAIA strategy across projects.

**Results:**

Based on this review and clarification of the operational definitions of the components of the SAIA, the four components of SAIA were mapped to 13 ERIC strategies. SAIA strategy meetings encompassed external facilitation, organization of provider implementation meetings, and provision of ongoing consultation. Cascade analysis mapped to three ERIC strategies: facilitating relay of clinical data to providers, use of audit and feedback of routine data with healthcare teams, and modeling and simulation of change. Process mapping matched to local needs assessment, local consensus discussions and assessment of readiness and identification of barriers and facilitators. Finally, continuous quality improvement encompassed tailoring strategies, developing a formal implementation blueprint, cyclical tests of change, and purposefully re-examining the implementation process.

**Conclusions:**

Specifying the components of SAIA provides improved conceptual clarity to enhance reproducibility for other researchers and practitioners interested in applying the SAIA across novel settings.

**Supplementary Information:**

The online version contains supplementary material available at 10.1186/s43058-023-00390-x.

Contributions to the literature SAIA is broadly adaptable to QI efforts involving complex, multi-step processes within health systems, in resource-limited as well as higher-resourced settings. SAIA is well-suited to quality improvement efforts in systems containing a defined care cascade and routinely available data, especially when modifications to workflows are within HCW control. Specification of the SAIA components provides improved conceptual clarity to enhance reproducibility for other researchers and practitioners interested in applying the SAIA across heterogeneous settings.

## Background

The field of implementation science (IS) focuses on improving the delivery of evidence-based interventions (EBI) to maximize their potential impact across heterogeneous settings. Implementation strategies, defined as methods or techniques employed to improve adoption, implementation, and sustainment of a clinical program or practice [1], are a major focus of the field. As the IS field has developed, generating evidence on effectiveness of implementation strategies to improve the delivery of EBIs across varied contexts has been a focus. Implementation strategies are key in guiding how to effectively realize EBIs in practice settings. In order to build the evidence base on implementation strategies, including how well they work across varied contexts, it is important for researchers to explicitly define and report on the core (essential) elements of implementation strategies.

Unclear terminology or inconsistent specification of implementation strategies has made replication of study findings in novel settings difficult [2–5]. Guidelines for naming, defining, and operationalizing implementation strategies have been proposed by Proctor et al. [2] to make explicit how others can use and adapt implementation strategies to novel settings, in order to further the science, disseminate more generalizable knowledge, and add conceptual clarity. These guidelines established seven dimensions of nomenclature: actor, action, action targets, temporality, dose, implementation outcomes addressed and theoretical, empirical, or pragmatic justification.

The Expert Recommendations for Implementing Change (ERIC) are another effort to create “a common nomenclature for implementation strategy terms, definitions and categories that can be used to guide implementation research and practice” [5] across heterogeneous health service settings. The ERIC expert panel reached consensus on 73 implementation strategies [6], whose use helps improve conceptual clarity, relevance, and comprehensiveness when reporting on implementation strategies.

The Systems Analysis and Improvement Approach (SAIA) is an evidence-based, multi-component implementation strategy focused on optimizing service delivery cascades [7]. SAIA combines systems engineering tools into an iterative process to guide service delivery staff and managers to visualize treatment cascade drop-offs and prioritize areas for system improvements, identify modifiable organization/facility-level bottlenecks, and propose, implement and assess the impact of modifications to improve system performance [8]. The core systems tools that the SAIA harnesses are cascade analysis [9] (whereby routine data is used to assess how the client population passes through specific sequential steps, identify drop off among the clients and prioritize steps for quality improvement efforts) [10], process mapping (where frontline service providers and managers collaboratively outline the steps that clients currently go through to achieve care in their specific organization/facility), and continuous quality improvement (CQI) [11–14], to guide service provider-led, data-driven quality improvement. This work is conducted through organization/facility-level learning meetings supported by external facilitators and conducted at set intervals, typically monthly, for a minimum of 6 months, to allow service providers to gain expertise in implementing SAIA to improve outcomes of their specific service. SAIA has been adopted across a range of geographic and clinical settings. The SAIA trial (PI: Sherr) tested SAIA through a 36-facility, cluster randomized trial in three SSA countries in prevention of mother-to-child transmission of HIV services [8]. The intervention led to *3.3-fold greater improvement in antiretroviral uptake for HIV-infected pregnant women* (13.3% vs 4.1%; increase to 77.7% in intervention and 65.9% in control facilities) and over *17-fold greater improvement in early infant diagnosis in HIV-exposed infants* (11.6% vs 0.7%; increase to 46.1% in intervention and 32.0% in control facilities) [7].

While care cascades have gained increasing traction as a useful way to organize data to inform actions, there are few implementation strategies using and optimizing care cascades that are tailored for LMIC and low-resourced settings. Most strategies target a single step in a system, whereas SAIA focuses on the system as a whole. In addition, the use of CQI ensures the contextual relevance of the proposed solutions to identified bottlenecks. SAIA’s added value relative to CQI stems from the addition of tools to encourage systems thinking among front line care providers and quantitative and qualitative prioritization techniques which use local data sources, prior to CQI solution generation. Over the last decade, there has been a steady rise in funded research to adapt SAIA to novel clinical areas and geographic settings and a growing demonstration of its broader effectiveness across a range of public health settings [15–21]. To extend on this previously published research and ensure SAIA’s success, its adaptation and implementation should be guided by conceptually clear implementation strategies. In this short report, we comprehensively map the core components of the SAIA implementation strategy to the distinct strategies of the ERIC typology, specify each resultant ERIC implementation strategy according to Proctor’s guidelines for specifying and reporting implementation strategies, and describe implementation outcomes that link to the multi-component SAIA strategy. By empirically and theoretically justifying the inclusion of each component of SAIA, we hope to make clear that CQI must be data driven, and should occur via supporting data use by care providers and support team’s systems thinking and prioritization skills [9, 22].

## Methods

Soliciting collective input to specify implementation strategies has been called for by leaders in the field of implementation science [2, 6, 23], in particular as the evidence-base on strategies like SAIA is rapidly emerging. To capture structured feedback and support consensus building, the investigators convened a panel of 23 implementation scientists, researchers, implementing team members, and organizational stakeholders, all with direct experience implementing and/or evaluating SAIA. This panel included those experienced with SAIA’s adaptation and application across a range of clinical areas (including PMTCT [8, 18, 19], mental health [16], hypertension [17], family planning [15], pediatric HIV [20], cervical cancer, community-based naloxone distribution [24], juvenile justice health care services, and malaria), and countries (Mozambique, Kenya, USA, Democratic Republic of the Congo), whose direct implementation experience made them well-suited to synthesize best practices and priorities for further adaptation and spread.

### Process

As pre-work, a smaller group of IS experts, engaged in the initial SAIA studies targeting the optimization of prevention of mother-to-child transmission of HIV (PMTCT) programs, convened to specify the components of the SAIA strategy (SAIA strategy meetings, cascade analysis, process mapping, CQI) and discuss the process by which a broader SAIA panel would be engaged. Subsequently, over 12 months, a modified nominal group technique approach (mNGT) [25] was employed to name, define, and operationalize SAIA core components using Proctor’s recommendations [2] and match them to ERIC implementation strategies. Three in-person meetings were held and multiple drafts reviewed to specify the actors, action, action targets, temporality, dose, implementation outcomes, and theoretical justification for each of the SAIA intervention components. Each component was presented independently followed by interactive debate, to gain consensus on the most appropriate mapping to ERIC strategies. Broader conversation across clinical areas highlighted commonalities and differences, clarifying the essential SAIA components, as well as broader linkages of this multi-component strategy to Proctor’s implementation outcomes [26]. Through consensus, the broader SAIA panel determined which Proctor implementation outcomes are effectively addressed through the use of the SAIA implementation strategy, a process that was informed by the published results of the various studies in peer reviewed journals [7, 9, 15, 16, 19, 20, 27] and conferences [24, 28] as well as feedback from field-based research teams. For example, the more recent adaptation of SAIA to optimize community-based Naloxone distribution in Oakland, California, provided a different setting from the remaining SAIA studies which have been primarily health facility-based. The mNGT sessions brought this issue to the group, and more inclusive language was adopted, replacing the terms patients and health care workers with clients and service providers. Implementation outcomes were considered for SAIA as a whole (not its individual components), as to date the multi-component strategy has been implemented holistically and mechanisms of action and contributions of individual components have not been assessed. The SRQR reporting guideline checklist was deemed appropriate for this short report and is available as an additional file (Additional file 1).

## Results

The components of the SAIA implementation strategy were named and operationally defined to guide further specification.

### Component 1

*SAIA Strategy Meeting* is defined as an assembly convened of frontline service providers by an external facilitator with expertise in SAIA. These 1–2 h long meetings usually occur monthly and the aforementioned external facilitators provide ongoing support and/or feedback on SAIA implementation to the service delivery team, including by guiding teams to operationalize micro-interventions and assign tasks and providing feedback on the appropriateness of a proposed micro-intervention to the cascade step or bottleneck it is intended to address.

### Component 2

*Cascade Analysis* is defined as use of a Cascade Analysis Tool (CAT, Additional file 2a) to analyze the implementing unit’s data, assess current performance of a multi-step care cascade, identify gaps, and quantify potential improvement to the system if a given step were optimized [9, 29, 30].

### Component 3

*Process Mapping* is when frontline service providers visualize, on paper, the service they are providing from the perspective of the target client population and identify bottlenecks and inefficiencies. Through the resulting process map, service providers discuss modifiable system challenges and then pair the results with the CAT optimization, to identify the step and/or target of future improvement efforts (Additional file 2b).

### Component 4

*CQI* is defined as using the results of the CAT and process mapping to propose and prioritize potential micro-interventions (modifications to workflow or service organization that are within the frontline provider’s power to influence, also referred to as small tests of change) [31] targeting the specific cascade step and/or service bottleneck identified. The micro-intervention is operationalized in terms of its goal, scope, timeframe, specific tasks, and responsible party. Once micro-interventions are identified for testing, their impact is assessed through the plan-do-study-act cycle [32]. At each SAIA strategy meeting, the implementation fidelity and impact of the micro-intervention are assessed and the decision is made to adopt, adapt, or abandon it (Additional file 2c).

Further details on the operationalization of the SAIA components are described in an additional file (Additional file 3) and is also available at www.saia-strategy.com.

Each of these four SAIA components was mapped to distinct ERIC implementation strategies by the broader research team, followed by specification of their strategy-specific actor(s), action(s), action target(s), temporality, dose, and intended implementation outcome(s) [2]. All results are presented in Table 1.

**Table 1:**
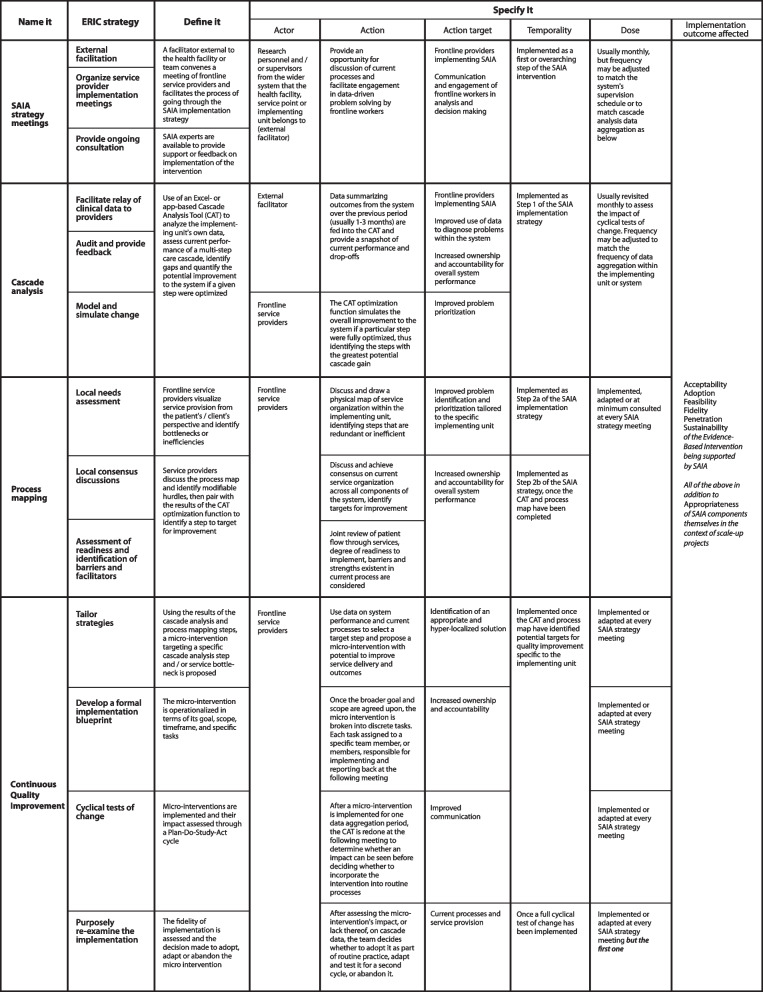
Specification of ERIC strategies contained within SAIA

#### SAIA strategy meetings

The action taken through SAIA strategy meetings is the creation of a discussion space of current processes, enabling engagement with data driven problem solving by the frontline service providers with support from external facilitators. The targets of this action are the frontline service providers implementing SAIA which may include those directly involved in the targeted service delivery or those tangentially impacted by the services (for example laboratory or pharmacy services). The SAIA strategy meetings are the venue through which the three remaining components of the SAIA implementation strategy are shared and discussed. The timing and periodicity of meetings can be adjusted to match the timing of supervision visits, availability of routine data, or other driving considerations at the site level.

#### Cascade analysis

Cascade analysis is accomplished in SAIA through the CAT [9, 29]. Sequentially linked, summarized outcome data from the site over a previous period (typically 1–3 months) is fed into the CAT and provides the team with a snapshot of current performance, including drop offs across steps. The optimization function simulates the overall improvement to the system if a particular step were fully optimized (assuming other steps remain constant), thereby identifying the steps with the greatest potential for cascade gain. The action targets of the cascade work are the frontline providers whose improved use of data to diagnose problems within the system bolsters their sense of ownership and accountability for overall performance. Cascade analysis is seen as the initial step of SAIA and is typically revisited monthly to assess the impact of CQI’s cyclical tests of change; however, frequency can be adjusted to match the frequency of data aggregation within the unit or systems’ health management information system (HMIS).

#### Process mapping

Process mapping facilitates the discussion and drawing of a physical map of how clients pass through services within the implementing unit, highlighting steps that are redundant, represent barriers or otherwise do not add value to the individual [33, 34]. Through reviewing these maps, teams discuss and achieve consensus on current service organization across all components of the system, while identifying target areas for improvement. The target is to improve problem identification and prioritization that is tailored to the specific implementing organization or unit. Process mapping, like cascade analysis, also reinforces ownership and accountability for system performance among the frontline team. Process mapping is the second step of SAIA and can be understood as a two-part step, whereby the first is the physical mapping and the second is local consensus discussions conducted after the CAT and process maps are completed [19]. At a minimum, process mapping is conducted once, at the first SAIA strategy meeting, but may be revisited and reworked as often as monthly throughout the implementation period.

#### CQI

The specific actions for this component are fourfold. First, health care teams use data on systems performance and current processes to select a target step and propose a micro-intervention with potential to improve service delivery and outcomes. Once the broader goal and scope are agreed upon, the micro-intervention itself is delineated into discrete tasks, each clearly assigned to a specific team member or members for implementation and reporting at the subsequent meeting. Once the micro-intervention has been implemented for the aggregated data period, the CAT is repeated to determine whether to integrate the micro-intervention into routine processes. After assessment of the intervention’s impact on cascade performance, the team decides together whether to adopt it as part of routine performance, adapt it and test it for a second cycle, or abandon it. The action targets of CQI include current processes and service provision as well as communication among the health care team. All actions are repeated at every SAIA meeting, with the exception of review of micro-interventions to adopt, adapt, or abandon, which only occurs after the initial SAIA strategy meeting. See Table 2 for example micro-interventions from SAIA projects.

**Table 2:** Example micro-interventions from SAIA projects

SAIA project, setting, and service area	Identified problem	Micro-intervention	Specific tasks
SAIA 3-country study, Mozambique, Kenya and Côte d’Ivoire Prevention of mother-to-child transmission of HIV	Laboratory delays are causing delays in treatment initiation	Increase communication between clinic and laboratory staff	[Named ANC nurse] will call [named laboratory technician] daily to check on results and make sure the report will be sent by Friday
SAIA-SCALE, Mozambique Prevention of mother-to-child transmission of HIV	Low proportion of HIV-exposed infants are entering child-at-risk care (CCR)	Monitor delivery ward–CCR linkage log daily	[3 named CCR nurses] will review the linkage log daily to identify eligible mother-infant pairs and make reminder phone calls
SAIA-SCALE, Mozambique Prevention of mother-to-child transmission of HIV	Low proportion of HIV-exposed infants are entering child-at-risk care (CCR)	Involvement of male partners to encourage uptake	[2 named ANC nurses] will extend partner invites for education on CCR importance to pregnant patients in ANC
SAIA-MH, Mozambique mental health	Patients are not returning after initial diagnosis	Outreach to new patients	[Service provider] will call patients 3 days before follow-up visit is due
SAIA-FP, Kenya HIV testing in family planning	Clients are declining testing in the family planning clinic	Improve counseling so clients recognize the benefit of testing	Health talks will be held at clinic to improve client sensitization

#### Implementation outcomes linked to SAIA

Implementation outcomes, defined as the effects of deliberate and purposive actions to implement new treatments, practices or services have three key functions: (1) they serve as indicators of implementation success, (2) they are proximal indicators of implementation processes, and (3) they are important intermediate outcomes [26, 35]. According to the broader SAIA research team whose perspective was informed by the published evidence of the existing SAIA studies, the multi-component implementation strategy of SAIA mapped to six of Proctor’s implementation outcomes [26]: *acceptability*, *adoption*, *feasibility*, *fidelity*, *penetration*8 and *sustainability*. The team identified these implementation outcomes to be related to the EBI that SAIA was designed to optimize (e.g., in adapting and effectiveness of SAIA for novel care cascades). However, as the focus of SAIA-related research moves from establishment of SAIA’s effectiveness for novel care cascades to testing strategies to spread and/or sustain SAIA, the implementation outcomes of interest expand to include *appropriateness* and *cost*, and focuses on improving implementation of SAIA as an evidence-based implementation strategy (as well as the EBI under study) (see Table 1). Operational definitions within the context of SAIA, as well as tools and approaches to measure each outcome are available in Additional file 3.

## Discussion

Based on this review and clarification of the operational definitions of the components of SAIA, the panel of experts mapped the ERIC strategies to each of the four SAIA components. SAIA mapped to 13 distinct ERIC strategies and as a multi-component implementation strategy aimed to impact six implementation outcomes: acceptability, adoption, appropriateness, feasibility, and penetration.

Frontline service providers are actors in the context of some SAIA component and action targets in others. As implemented to date, SAIA relies on an external facilitator to convene meetings and guide teams through SAIA implementation. Fidelity to SAIA facilitation is tracked through routine monitoring and its quality is assessed through periodic qualitative assessment using an implementation science determinants framework, such as the Consolidated Framework for Implementation Research (CFIR) [19, 27]. The completed SAIA trials have been implemented by study nurses as facilitators. Ongoing SAIA trials are experimenting with other types of study facilitators include social workers, community health workers, mental health technicians, and medical doctors. Sustainability may require the external facilitator to eventually be phased out, and a facilitator to instead be assigned directly from existing management structures, such as sub-national agencies, already tasked with organization/facility oversight and support, an approach that was assessed in the scale-up study of SAIA for PMTCT [18], or transition to facilitation by a champion among the frontline service providers themselves.

SAIA is adaptable to a variety of care cascades and contexts. Our current work aims to facilitate future adaptations while maintaining reproducibility. Specific work exploring mechanisms of action (and the relative contributions of individual components of SAIA) is underway and will build upon the generalizability of the SAIA, including through the use of longitudinal structural equation modeling [36].

Of note, existing data on the service implementing the target EBI, which are key to data-driven systems-level thinking on current performance, varied across settings in its availability or accessibility. This required some study teams to work with key stakeholders (Ministry of Health, others) to introduce or add to data collection forms, or to develop creative ways to collate data across multiple data sources. This is particularly critical for the cascade analysis component. Given that many settings in which SAIA is being implemented are transitioning from acute to chronic care systems, this is hardly surprising. Service providers are being tasked to not just generate and supply data ‘up the chain of command’ but use it to identify bottlenecks and generate solutions for their systems. Thus, the initial work of SAIA often addresses the perennial challenge of data use by frontline service providers for decision-making [37].

## Conclusions

SAIA represents a promising approach to harness systems-level knowledge of service providers and managers at the frontline of care, both in clinical and community settings. In order to ensure its successful and accurate translation to other clinical areas and geographic regions, the authors have built upon a growing body of SAIA research by detailing its core components and implementation strategies, through use of established specification approaches. This work provides clear definitions of the SAIA components using established taxonomy, and maps the SAIA strategy to implementation outcomes they may activate, in order to facilitate future adaptations and additionally lay the groundwork for future work to define its mechanisms of action.

## Supplementary Information

**Additional file 1.** SRQR Reporting guideline checklist.

**Additional file 2.** a: Cascade Analysis Tool (CAT). b: Process mapping guide. c: Continuous Quality Improvement (CQI) action planning tool and instructions.

**Additional file 3.** Operational Definitions of SAIA.

## Data Availability

Data sharing is not applicable to this article as no datasets were generated or analyzed during the current study.

## Abbreviations

CAT: Cascade Analysis Tool
EBI: Evidence-based intervention
ERIC: Expert Recommendations for Implementing Change
HCW: Health care worker
HMIS: Health management and information system
IS: Implementation science
PMTCT: Prevention of mother-to-child transmission of HIV
QI: Quality improvement
SAIA: Systems Analysis and Improvement Approach
