# Correction: The Systems Analysis and Improvement Approach: specifying core components of an implementation strategy to optimize care cascades in public health

**DOI:** 10.1186/s43058-023-00418-2

**Published:** 2023-03-27

**Authors:** Sarah Gimbel, Kristjana Ásbjörnsdóttir, Kristin Banek, Madeline Borges, Jonny Crocker, Joana Coutinho, Vasco Cumbe, Aneth Dinis, McKenna Eastment, Douglas Gaitho, Barrot H. Lambdin, Stephen Pope, Onei Uetela, Carmen Hazim, R. Scott McClelland, Ana Olga Mocumbi, Alberto Muanido, Ruth Nduati, Irene N. Njuguna, Bradley H. Wagenaar, Anjuli Wagner, George Wanje, Kenneth Sherr

**Affiliations:** 1 Department of Child, Family, and Population Health Nursing, University of Washington, Magnuson Health Science Bldg, Seattle, WA USA; 2 Department of Global Health, University of Washington, Seattle, WA USA; 3 Center for Public Health Sciences, University of Iceland, Reykjavík, Iceland; 4 Department of Epidemiology, University of Washington, Seattle, WA USA; 5 Institute for Global Health and Infectious Diseases, University of North Carolina, Chapel Hill, NC USA; 6 Comité Para a Saúde de Moçambique, Beira, Mozambique; 7 Ministry of Health, Provincial Health Department, Sofala, Mozambique; 8 Ministry of Health, National Department of Public Health, Maputo, Mozambique; 9 Department of Medicine, University of Washington, Seattle, WA USA; 10 Network of AIDS Researchers of East and Southern Africa, Nairobi, Kenya; 11 RTI International, Berkeley, CA USA; 12 Instituto Nacional de Saúde de Maputo, Maputo, Mozambique; 13 Universidade Eduardo Mondlane, Maputo, Mozambique; 14 University of Nairobi, Nairobi, Kenya; 15 Research and Programs, Kenyatta National Hospital, Nairobi, Kenya; 16 Department of Medical Microbiology, University of Nairobi, Nairobi, Kenya

**Correction** **:** **Implement Sci Commun 4, 15 (2023)**

**https://doi.org/10.1186/s43058-023-00390-x**

Following publication of the original article [1], an error was identified in the Background section.

There is a duplicated paragraph, the original article has now been updated to remove the duplicate and read the following:

“The Systems Analysis and Improvement Approach (SAIA) is an evidence-based, multi-component implementation strategy focused on optimizing service delivery cascades [7]. SAIA combines systems engineering tools into an iterative process to guide service delivery staff and managers to visualize treatment cascade dropoffs and prioritize areas for system improvements, identify modifiable organization/facility-level bottlenecks, and propose, implement and assess the impact of modifications to improve system performance [8]. The core systems tools that the SAIA harnesses are cascade analysis [9] (whereby routine data is used to assess how the client population passes through specific sequential steps, identify drop off among the clients and prioritize steps for quality improvement efforts) [10], process mapping (where frontline service providers and managers collaboratively outline the steps that clients currently go through to achieve care in their specific organization/facility), and continuous quality improvement (CQI) [11–14], to guide service provider-led, data-driven quality improvement. This work is conducted through organization/facilitylevel learning meetings supported by external facilitators and conducted at set intervals, typically monthly, for a minimum of 6 months, to allow service providers to gain expertise in implementing SAIA to improve outcomes of their specific service. SAIA has been adopted across a range of geographic and clinical settings. The SAIA trial (PI: Sherr) tested SAIA through a 36-facility, cluster randomized trial in three SSA countries in prevention of mother-to-child transmission of HIV services [8]. The intervention led to *3.3-fold greater improvement in antiretroviral uptake for HIV-infected pregnant women* (13.3% vs 4.1%; increase to 77.7% in intervention and 65.9% in control facilities) and over *17-fold greater improvement in early infant diagnosis in HIV-exposed infants* (11.6% vs 0.7%; increase to 46.1% in intervention and 32.0% in control facilities) [7].”

Following the publication of the original article [1] the authors requested to update the “Competing interests” section as follows:

“The authors declare that Sarah Gimbel and Kenneth Scherr are members of the Editorial Board for the Journal of Implementation Science Communications.”

The original article [1] has been corrected.
