# Supplementary material for: The Systems Analysis and Improvement Approach: specifying core components of an implementation strategy to optimize care cascades in public health
Source: Implement Sci Commun. 2023 Feb 14;4:15. doi: 10.1186/s43058-023-00390-x (PMC9926643; doi:10.1186/s43058-023-00390-x)
Supplement: Supplementary file 1 — Additional file 1. SRQR Reporting guideline checklist. [file 43058_2023_390_MOESM1_ESM.pdf]

SRQR table for SAIA Specification

Topic	Item
Title	**The Systems Analysis and Improvement Approach (SAIA): Specifying core components of an implementation strategy to optimize care cascades in public health**
Abstract	**Background:** The Systems Analysis and Improvement Approach (SAIA) is an evidence-based, multi-component implementation strategy that engages service providers in the use of routinely-available service data to optimize service delivery cascades and promote systems-level thinking. SAIA was originally developed to address bottlenecks in HIV care in low-and middle-income countries, but has since been adapted and applied to a variety of care systems including: cervical cancer screening, mental health treatment, hypertension management, family planning, and community-based naloxone distribution. These projects have been implemented across a variety of settings in sub-Saharan Africa and the United States. Given the diversity of implementation experience, our consortium aimed to define the core elements of SAIA, to improve reproducibility, guide future adaptations, and lay the groundwork to evaluate mechanisms of action.<br><br>**Methods**: Specification of the SAIA strategy was undertaken over 12 months by an expert panel of SAIA researchers using a three-round, modified nominal group technique approach to match core SAIA components to the Expert Recommendations for Implementing Change (ERIC) list of distinct implementation strategies. Core implementation strategies were then specified according to Proctor's recommendation for specifying and reporting, followed by consensus on related implementation outcomes that link to the multi-component SAIA strategy.<br><br>**Results:** The four components of the SAIA strategy: (1) SAIA strategy meetings; (2) cascade analysis; (3) process mapping; and (4) continuous quality improvement, mapped to 13 distinct ERIC strategies. The SAIA strategy meetings component mapped to external facilitation, organization of provider implementation meetings, and provision of ongoing consultation. Cascade analysis mapped to facilitating relay of clinical data to providers, use of audit and feedback, and modelling and simulation of change. Process mapping tied to local needs assessment, local consensus discussions, and assessment of readiness and identification of barriers and facilitators. Continuous quality improvement encompassed tailoring strategies, developing a formal implementation blueprint, cyclical tests of change and purposefully re-examining the implementation process.<br><br>**Conclusions**: Formally specifying the core components of SAIA provides improved conceptual clarity to enhance reproducibility for other researchers and practitioners interested in applying the SAIA across novel settings. Furthermore, this work provides a structured framework to examine potential mechanisms of SAIA and its component implementation strategies.
**Introduction**	

SRQR table for SAIA Specification

Problem formulation	SAIA was originally developed to address bottlenecks in HIV care in low- and middle-income countries, but has since been adapted and applied to a variety of care systems across a variety of settings in sub-Saharan Africa and the United States. Given the diversity of implementation experience and the potential for future adaptations, it is important that SAIA be guided by clear implementation strategies and specified according to established guidelines.
Purpose or research question	Researchers with experience implementing SAIA across diverse care systems set out to specify SAIA in two standardized ways: using the guidelines proposed by Proctor *et al* and the Expert Recommendations for Implementing Change (ERIC). In addition, the multi-component implementation strategy was linked to implementation outcomes.
**Methods**	
Qualitative approach and research paradigm	A modified nominal group technique approach was employed over a period of 12 months, to name, define and operationalize the core components of SAIA using Proctor's guidelines, and the core components were matched to relevant ERIC strategies.
Researcher characteristics and reflexivity	Researcher had direct experience implementing the SAIA. Specifically, they led or were co-investigators or managed SAIA projects.
Context	In-person (at the University of Washington) and virtual collaboration were the settings for this work that included participants in Europe, sub-Saharan Africa and the United States.
Sampling strategy	A panel of 23 implementation science researchers was convened to participate in the work. Panelists were purposively selected to include researchers with expertise in SAIA and direct involvement in projects forming part of the SAIA consortium of funded research.
Ethical issues pertaining to human subjects	N/A
Data collection methods	A series of panel discussions was conducted to gain full consensus on specification of SAIA. All panel members had veto power during the debate. Conclusions were recorded during the panel discussions and shared with all members for review following each meeting.
Data collection instruments and technologies	We used Zoom to hold joint meetings and reviews of drafts and summaries were shared via email.
Units of study	N/A
Data processing	N/A
Data analysis	
Techniques to enhance trustworthiness	Multiple rounds of joint discussion and ample time for individual and joint feedback on findings and drafts until consensus was achieved.
Results / findings	The SAIA implementation strategy components were defined as 1) *SAIA Strategy Meetings*; (2) *Cascade Analysis*; (3) *Process Mapping*; and (4) *Continuous Quality Improvement (CQI)*. SAIA components were mapped to 13 ERIC strategies: (1) External facilitation, organization of implementation meetings and provision of ongoing consultation; (2) facilitating relay of clinical data to providers, use of audit and feedback and modeling and

	simulating change; (3) local needs assessment, local consensus discussions, assessment of readiness and identification of barriers and facilitators and (4) conducting cyclical small tests of change, development of a formal implementation blueprint, tailoring of implementation strategies and purposefully re-examining the implementation process. SAIA mapped to six of Proctor's implementation outcomes as they relate to supporting the application of evidence-based interventions, including *acceptability, adoption, feasibility, fidelity, penetration* and *sustainability*.
Synthesis and interpretation	
Links to empirical data	
**Discussion**	
Integration with prior work, implications, transferability, and contribution(s) to the field	Formally specifying the core components of SAIA provides improved conceptual clarity to enhance reproducibility for other researchers and practitioners interested in applying the SAIA across novel settings. Furthermore, this work provides a structured framework to examine potential mechanisms of SAIA and its component implementation strategies.
Limitations	
**Other**	
Conflicts of interest	None noted.
Funding	This work was supported from grants from the National Institutes of Health, including R01MH113435 (SAIA-SCALE), F32HD088204 and R34AI129900 (SAIA-PEDS), R21AI124399 (mPCAT), K24HD088229 (SAIA-FP), R21MH113691 (SAIA-MH), P30AI027757 (CFAR), R21DA046703 (SAIA-Naloxone), R01HL142412 (SAIA-HTN), 1UG3HL156390-01 (SCALE SAIA-HTN) R01HD0757 and R01HD0757-02S1 (SAIA), K08CA228761 (CCS SAIA) and T32AI070114 (UNC TIDE), Support was provided by the Implementation Science Core of the University of Washington/Fred Hutch Center for AIDS Research, an NIH-funded program under award number AI027757 which is supported by the following NIH Institutes and Centers: NIAID, NCI, NIMH, NIDA, NICHD, NHLBI, NIA, NIGMS, and NIDDK. This work was also supported by the Doris Duke Charitable Foundation and the Rita and Alex Hillman Foundation (SAIA-JUV), and the Thrasher Foundation (SAIA-MAL).
