# Supplementary material for: The Systems Analysis and Improvement Approach: specifying core components of an implementation strategy to optimize care cascades in public health
Source: Implement Sci Commun. 2023 Feb 14;4:15. doi: 10.1186/s43058-023-00390-x (PMC9926643; doi:10.1186/s43058-023-00390-x)
Supplement: Supplementary file 2 — Additional file 2. a: Cascade Analysis Tool (CAT). b: Process mapping guide. c: Continuous Quality Improvement (CQI) action planning tool and instructions. [file 43058_2023_390_MOESM2_ESM.zip › additional file 2/Additional File 2c.pdf]

SAIA TOOL & USER GUIDE FOR CQI ACTION PLANNING

Instructions:
This form is used by district supervisors to orient the action planning and CQI discussions with the health facility team during their monthly SAIA Strategy Meetings. Discussion points in this form correspond to the columns in the *CQI ACTION PLANNING TOOL.*

Discussion guide to identify problems and plan solutions (micro-intervention)

INTRO AND COLUMNS A-B: Write the name of the district, the facility, the date, and round number

COLUMN C: On reviewing the CAT tool what are the steps of the CAT that can be improved? Choose one of the steps to prioritize and list it.

COLUMN D: What is the baseline value and percentage achieved of the prioritized CAT step at this health facility?

COLUMN E: Describe the problems associated with the prioritized CAT step listed in column C. Consult the process map to orient the discussion. Which of these problems is the most important to discuss?

- Once the problem is selected, ask someone to write the "identified problem" in column E.

COLUMN F: In what service point at the facility is this problem most relevant?

- Once the service point is agreed upon, write it in column F.

COLUMN G: Discuss the possible solutions for the problem identified. Select just one solution that will best resolve the identified problem, and describe this solution. Include the actions (or what will be done?), objectives (why this is necessary?), fundamentals (why this work will be important?). Be specific.

- List the proposed solution in column G.

Examples
- *Improve coordination between the laboratory and clinical services.*
- *Streamline services for families.*

COLUMN H: Plan the solution (micro-intervention). When will it start? What specific tasks or duties should be accomplished?

- Enter the tasks and assignments in column H.

Examples
- *Weekly communication with the laboratory to identify certain results missing client ID numbers*
- *Asking patients when other family members are scheduled to come for services and then creating one time/date for the family to ease travel*

COLUMN I: Who will be responsible for each task and duty?

- List the team or individuals responsible in column I.

Examples
- *Team lead – Joana*
- *Nurse - Melita*

COLUMN J: How will you know if there has been an improvement? What do you expect will occur?

- List the expected outcome in column J.

Examples
- *Decrease wait times for testing*
- *Decrease the number of required visits for clients*
- *Increase the number of clients served*

Monitoring guide for previous months solutions

One month after the SAIA strategy meeting where a solution (micro-intervention), duties and tasks have been assigned, use the following questions to start the discussion with the facility team.

COLUMN K: Was the planned solution (micro-intervention) actually implemented?

- Note the response with in the "was the micro-intervention implemented" column K.

Options
- *Yes (was implemented as planned)*
- *No (was not implemented)*
- *Partly (was implemented but not completely or correctly)*

COLUMN L: What changed in the last month as a result of this solution (micro-intervention)?

- Refer to column C to remember what CAT step was selected
- Note the new value of the relevant CAT step in column L.

COLUMN M: What tasks and assignments were completed?

- Refer to column H to remember was tasks and assignments were agreed to.
- Note whether they were completed or not (Yes, No, Partly) in column M.

COLUMN N: What will you do with this solution (micro-intervention) in the future? Discuss whether it should be abandoned, adapted or adopted.

- Note abandon, adapt, or adopt in column N.

Fill in while planning the micro-intervention										Fill in one month later			
A	B	C	D	E	F	G	H	I	J	K	L	M	N
Date	*Round*	*Targeted CAT step*	*Baseline value (#/%) of CAT step*	*Problems identified*	*Service point*	*Solution (micro-intervention)*	*Tasks and Assignments*	*Team/ individuals delegated*	*Anticipated change*	*Was the micro-intervention implemented (yes, partly, no)?*	*Value (#/%) of CAT step at 1 month*	*Which assignments and tasks were completed?*	*Do you plan to adopt, adapt or abandon the micro-intervention?*
