# Supplementary material for: The Systems Analysis and Improvement Approach: specifying core components of an implementation strategy to optimize care cascades in public health
Source: Implement Sci Commun. 2023 Feb 14;4:15. doi: 10.1186/s43058-023-00390-x (PMC9926643; doi:10.1186/s43058-023-00390-x)
Supplement: Supplementary file 2 — Additional file 2. a: Cascade Analysis Tool (CAT). b: Process mapping guide. c: Continuous Quality Improvement (CQI) action planning tool and instructions. [file 43058_2023_390_MOESM2_ESM.zip › additional file 2/Additional File 2b.pdf]

PROCESS MAPPING GUIDELINES

Participants: Frontline service providers and managers

Materials / Resources: Writing instrument (pen/pencil), paper, meeting space

STEP 1 - Select a care process to map

Decide which care process to map. Review whether this care process has been mapped before. If previously developed, work from that map – no need to recreate from scratch.

STEP 2 - Collect information and create a process map of current flow

To create a process map, start by interviewing at least 2 key informants (e.g., frontline healthcare workers, facility managers, receptionists, other healthcare personnel) who are knowledgeable about the process and client flow ***as it currently exists***. Take detailed notes.

Tip: In mapping out a care process, it is helpful to interview frontline healthcare workers who directly provide this care. It is important to get more than one perspective as, in many sites, healthcare workers operate or understand their systems differently.

Create a separate draft process map for each person interviewed. Use the appropriate process mapping symbols to the right to draw what is described. Drawings can be done by hand initially and then generated on a computer.

Tip: In collecting information and drawing the process map, tell the key informant to imagine you are a patient entering the care services. Together, you can walk through the clinic following the steps that patients in the care process take. You can document this process in real time with a clipboard and pencil. As you "walk the walk" of the patient, draw their steps, including where they move across the clinic to receive care, and the choices they make.

Helpful questions to ask include:

1. Where do I go first?
2. What happens here?
3. Who do I talk to (e.g., registration, triage)?
4. How long does this take?
5. What kind of data is collected?
6. How are my records stored and retrieved?
7. What type of identification number is used?
8. Where do I go next?

PROCESS MAPPING SYMBOLS

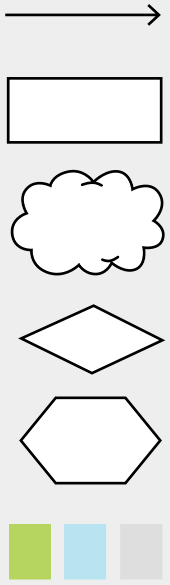

- **Next step**
- **Care cascade activity**
- **Wait/Travel time**
- **Branch point (Y/N, +/-)**
- **Endpoint**
- **Color coding for different services, staff, spaces**

Remember! First create a map of the **current process**, not the process as it should occur. This process should be from the perspective of the client, not the service provider.

SAIA
Systems Analysis & Improvement Approach

Once you feel your draft process map is complete, consider the client wait times shown for each step in the process. Mark each wait time as:

1. Wait time is reasonable
2. Wait time is slightly too long
3. Wait time is far too long

Consider which wait times are most concerning. Mark them on the flow map and add any additional comments or questions regarding the client cascade and wait times.

> ***Tip:*** A variation to consider as you go through these steps and consider client wait times, you can also consider the payer perspective of the clients at each step (i.e., when are the clients spending money?).

STEP 3 - Share, analyze, and synthesize the current state process map with stakeholder healthcare team members

Share the various current process maps across a larger group of stakeholders (e.g., local managers and frontline healthcare workers on the care teams at the healthcare facility). Review each step of the various process maps together and analyze for accuracy.

> ***Tip:*** You can transfer these maps to flip charts for easier viewing by all parties involved.

Use the following questions to guide development of a single, accurate map of how things are currently being done (not how they should be done).

- Do you see anything that looks incorrect or that needs to be changed?
- Do you see any steps that are missing and should be added?
- Is there redundancy in the pathway?
- Where are healthcare workers are doing steps differently?
- Are there any additional pathways along which clients can move, which are not included on this map?

> ***Tip:*** When there is disagreement about a step, encourage discussion to gain consensus on how the step most likely is operating.

STEP 4 - Identify and prioritize process steps and areas for improvement with stakeholder healthcare team

After you share and discuss the current state process map with the healthcare team, you must identify priority areas for improvement efforts. Together with the team rate each process step on the map as:

- Unambiguously adding value to the client
- Adding no value to the client, but are unavoidable
- Adding no value to the client, but are avoidable

By clarifying which process steps add no value to the client and which are avoidable, the team is more able to focus in on which steps should be prioritized for improvement via small tests of change. The group should then select their prioritized process step for improvement.

Once use of the cascade analysis tool and process mapping have been completed, the team is ready to conduct small tests of change via continuous quality improvement (CQI).
