# Supplementary material for: The Systems Analysis and Improvement Approach: specifying core components of an implementation strategy to optimize care cascades in public health
Source: Implement Sci Commun. 2023 Feb 14;4:15. doi: 10.1186/s43058-023-00390-x (PMC9926643; doi:10.1186/s43058-023-00390-x)
Supplement: Supplementary file 3 — Additional file 3. Operational Definitions of SAIA. [file 43058_2023_390_MOESM3_ESM.docx]

**Operational Definitions of SAIA**.

*Acceptability*, the perception among implementation stakeholders that a given treatment, service, practice or innovation is agreeable, palatable or satisfactory, is determined by the user’s knowledge or direct experience with the treatment, practice or service, being implemented. Without exception, SAIA was reported by stakeholders across user-levels as an adequate implementation strategy to increase the acceptability of evidence-based treatments, practices or services being targeted. An example is the pilot to assess acceptability and feasibility of SAIA for the mental health care cascade in Mozambique, where findings documented the strength of the SAIA implementation strategy emanating from its ability to provide healthcare staff with tools integrated into their existing workflows which allowed them to make their own systems-level modifications to improve the quality of care services provided^1^. Acceptability across SAIA adaptations is assessed via qualitative interviewing techniques (e.g. in-depth interviews and focus group discussions) with frontline service providers and managers, as well as external facilitating agents, after the intensive implementation phase.^2,3^ Interview guides are developed using the Consolidated Framework for Implementation Research.

*Adoption* is defined as the intention, initial decision or action to introduce an innovation or evidence-based practice. An example of SAIA supporting adoption of an EBI at the facility level comes from the SAIA-Family Planning intervention, which aimed to increase HIV testing and counseling in family planning clinics in Mombasa, Kenya. At the start of the project, two of the 11 participating clinics were conducting no HIV testing. Through SAIA cycles, teams at these clinics identified a lack of trained counselors as the primary barrier and were ultimately able to gain approval for and hire counselors, who supported adoption of integrated testing and counseling^4^. Many SAIA adaptations employ the Organization Readiness for Implementing Change (ORIC)^5^ as well as related facility-level readiness assessments tailored to context, to assess whether settings are more or less inclined to adopt SAIA itself as well as the target EBI. The ORIC tool is optimally administered soon after introduction of the SAIA, typically within one to three months after start. The health facility assessments are conducted initially at start up and then annually thereafter to gauge whether structural readiness (such as supply of needed equipment, materials, supplies, or other resources) foster adoption. These assessments can then be revisited at endline to determine if *a priori* readiness was associated with adoption.

*Feasibility*, the extent to which a new treatment, practice or service can be used successfully or employed within a specific setting, has been assessed retrospectively across the various SAIA adaptations. In a similar fashion as *acceptability*, the implementation outcome of *feasibility* has been evaluated through qualitative interviews with individuals and user groups, using a CFIR lens, after piloting and then again after intensive implementation. SAIA as an implementation strategy bolstered perceived feasibility among end users of target EBI across adaptations. An example of SAIA supporting feasibility of EBIs was observed in a pilot study of SAIA for optimizing hypertension care (SAIA-HTN), conducted in two facilities over 12 weeks in preparation for a cluster randomized trial. Although part of MOH guidelines, hypertension screening was not routinely implemented at the start of the pilot, with blood pressure measurement occurring at just 5% of outpatient visits (range 3-6%). In the 12 weeks of the intervention, screening increased to 36% (range 25-47%). Focus group discussions and in-depth interviews with frontline health workers and managers revealed their enthusiasm for SAIA-HTN, and appreciation for the timely and relevant focus on hypertension.^6^

*Fidelity*, defined as the degree to which an intervention was implemented as it was intended, has been captured across SAIA adaptations in two distinct ways. First, the degree to which the target EBI is implemented is addressed through monthly capture of both process and clinical outcomes (via the CAT). The targeted focus on the outcomes, both process and clinical, keep the service delivery teams focus on EBI implementation fidelity. For example, the SAIA pilot across four sites to optimize implementation of mental health services was associated with increased quality of care regarding patients returning for follow up visits on time, adherent to medication and with function improvement.^1^ In the first trial of SAIA for PMTCT in Côte d'Ivoire, Kenya, and Mozambique, initiation of antiretroviral therapy and early infant diagnosis in accordance with national guidelines improved significantly in intervention facilities, compared to control facilities. ^1,2,4,7-10^ Second, many SAIA adaptations monitor through regular reporting, how completely individual organizations/facilities implement the SAIA components as intended. Are cascades and process maps reviewed and updated routinely? Are results of CQI reviewed and revised and new ones implemented at SAIA strategy meetings at the prescribed interval? This is necessary to understand how well and systematically the SAIA implementation strategy is employed across the often heterogenous settings.

*Penetration*, defined as the integration of practice within a service setting and its subsystems^11,12^, was reported to be amplified by many SAIA adaptations. Because use of a SAIA implementation strategy involves service providers regularly reviewing both quantitatively and qualitatively how clients access care and treatment, their subsequent understanding of the target EBI individually and as a care team is deeper and more contextualized. An illustrative example is the SAIA-Naloxone pilot, whose results suggested it had strong potential for improving penetration of naloxone distribution from syringe service programs (SSP). SSPs in Oakland, California used SAIA to prioritize improved data collection procedures, proactively screen and identify naloxone naïve participants and streamline the naloxone refill systems. After the six month SAIA pilot, the weekly average of people receiving naloxone increased from 6 to 62, and a significant increase in the weekly average number of naloxone doses per week was also observed.^13^

*Sustainability*, the extent to which a newly implemented EBI is maintained or institutionalized into operations at a service site, is bolstered by the SAIA implementation strategy. The EBI’s implementation is reviewed, revisited, reimagined, and refined through the SAIA process by the service delivery team, and in most SAIA adaptations, considerable focus is paid to a maintenance phase, when study inputs are reduced, allowing the team to better understand drivers of the EBI implementation success (or failure) in more ‘real world’ settings. In the SAIA-SCALE project, a prospective evaluation design was used to determine the sustainability of SAIA intervention when external support is reduced or removed. Findings demonstrated a modest reduction in supervision frequency and an increase in staff participation during the maintenance phase^14^.

Two implementation outcomes, specifically, *appropriateness* and *cost*, were not seen as related to the SAIA’s support of EBI implementation. Appropriateness, the perceived fit, relevance or compatibility of the EBI for a given practice setting, provider or consumer; and/or the perceived fit of the innovation to address a particular issue or challenge, was rejected because the SAIA strategy itself does not modify the EBI to be more appropriate and it is uncommon for SAIA micro-interventions to focus on modifying perceptions of fit/relevance among service delivery team members or clients. SAIA focuses primarily on adoption and increasing acceptability of EBIs already supposed to be implemented. Rather than focusing on improving perceptions of fit, SAIA targets actually improving fit. Cost, defined as the cost impact of the implementation effort, was also excluded because the SAIA multi-component implementation strategy is not specifically designed to affect costs of EBI implementation. Most adaptations to SAIA which are being evaluated, however, do conduct concurrent economic evaluations of the approach to provide guidance to governments and other stakeholders who may be interested in scaling the work on a broader scale.

1. Fabian KE, Muanido A, Cumbe VFJ, et al. Optimizing treatment cascades for mental healthcare in Mozambique: preliminary effectiveness of the Systems Analysis and Improvement Approach for Mental Health (SAIA-MH). Health Policy Plan 2021;35:1354-63.

2. Gimbel S, Rustagi AS, Robinson J, et al. Evaluation of a Systems Analysis and Improvement Approach to Optimize Prevention of Mother-To-Child Transmission of HIV Using the Consolidated Framework for Implementation Research. J Acquir Immune Defic Syndr 2016;72 Suppl 2:S108-16.

3. Lambdin BH, Zibbell J, Wheeler E, Kral AH. Identifying gaps in the implementation of naloxone programs for laypersons in the United States. Int J Drug Policy 2018;52:52-5.

4. Eastment MC, Wanje G, Richardson BA, et al. Results of a cluster randomized trial testing the systems analysis and improvement approach to increase HIV testing in family planning clinics. AIDS 2022;36:225-35.

5. Shea CM, Jacobs SR, Esserman DA, Bruce K, Weiner BJ. Organizational readiness for implementing change: a psychometric assessment of a new measure. Implement Sci 2014;9:7.

6. Gimbel S. Get with the Flow: Optimizing Hypertension Care Cascades for People Living with HIV in Mozambique. Ignite for Aging. Seattle, WA: University of Washington, School of Nursing, deTornay Center for Health Aging 2018.

7. Wagner AD, Augusto, O., Njuguna, I.N., Gaitho, D., Mburu, N., Oluoch, G., Carimo, N., Mwaura, P., Cherutich, P., Oyiengo, L., Gimbel, S., John-Stewart, G.C., Nduati, R., Sherr, K. Systems analysis and improvement approach to optimize the pediatric and adolescent HIV cascade (SAIA-PEDS): a pilot study. Implementation Science Communications 2022.

8. Beima-Sofie K WA, Soi C, Liu W, Tollefson D, Njuguna IN, Awino E, Gaitho D, Mburu N, Oluoch G, Mwaura P, Cherutich P, Oyiengo L, John-Stewart GC, Nduati R, Sherr K, Gimbel S. Providing “a beam of light to see the gaps:” – Determinants of implementation of the Systems Analysis and Improvement Approach applied to the pediatric and adolescent HIV cascade in Kenya. Implementation Science Communications under review.

9. Rustagi AS, Gimbel S, Nduati R, et al. Implementation and Operational Research: Impact of a Systems Engineering Intervention on PMTCT Service Delivery in Cote d'Ivoire, Kenya, Mozambique: A Cluster Randomized Trial. J Acquir Immune Defic Syndr 2016;72:e68-76.

10. Wagner AD, Gimbel S, Asbjornsdottir KH, et al. Cascade Analysis: An Adaptable Implementation Strategy Across HIV and Non-HIV Delivery Platforms. J Acquir Immune Defic Syndr 2019;82 Suppl 3:S322-S31.

11. Stiles PG, Boothroyd RA, Snyder K, Zong X. Service penetration by persons with severe mental illness: how should it be measured? J Behav Health Serv Res 2002;29:198-207.

12. Proctor E, Silmere H, Raghavan R, et al. Outcomes for implementation research: conceptual distinctions, measurement challenges, and research agenda. Adm Policy Ment Health 2011;38:65-76.

13. Lambdin BH, Kral, A., Wagner, A., Wegner, L., Sherr, K. Optimizing naloxone distribution to prevent opioid overdose fatalities: Results from piloting the systems analysis and improvement approach within syringe service programs. Proceedings from the 13th Annual Conference on the Science of Dissemination and Implementation, . Washington, D.C.: Implementation Science; 2013:S-99.

14. Crocker J, Agostinho, M., Amaral, F., Asbjornsdottir, K.A., Coutingo, J., Cruz, E., Dinis, A., Gimbel, S., Inguane, C., Sherr, K. Measuring the m in the RE-AIM framework: Using a stepped wedge design to evaluate maintenance of the saia-scale PMTCT program post-external support in Mozambique. 13th Annual Conference on the Science of Implementation; 2021; Washington D.C.: Implementation Science. p. 49.
